# DA-LSTM-VAE: Dual-Stage Attention-Based LSTM-VAE for KPI Anomaly Detection

**DOI:** 10.3390/e24111613

**Published:** 2022-11-05

**Authors:** Yun Zhao, Xiuguo Zhang, Zijing Shang, Zhiying Cao

**Affiliations:** School of Information Science and Technology, Dalian Maritime University, Dalian 116026, China

**Keywords:** KPI anomaly detection, VAE, LSTM, attention mechanism, adaptive threshold

## Abstract

To ensure the normal operation of the system, the enterprise’s operations engineer will monitor the system through the KPI (key performance indicator). For example, web page visits, server memory utilization, etc. KPI anomaly detection is a core technology, which is of great significance for rapid fault detection and repair. This paper proposes a novel dual-stage attention-based LSTM-VAE (DA-LSTM-VAE) model for KPI anomaly detection. Firstly, in order to capture time correlation in KPI data, long–short-term memory (LSTM) units are used to replace traditional neurons in the variational autoencoder (VAE). Then, in order to improve the effect of KPI anomaly detection, an attention mechanism is introduced into the input stage of the encoder and decoder, respectively. During the input stage of the encoder, a time attention mechanism is adopted to assign different weights to different time points, which can adaptively select important input sequences to avoid the influence of noise in the data. During the input stage of the decoder, a feature attention mechanism is adopted to adaptively select important latent variable representations, which can capture the long-term dependence of time series better. In addition, this paper proposes an adaptive threshold method based on anomaly scores measured by reconstruction probability, which can minimize false positives and false negatives and avoid adjustment of the threshold manually. Experimental results in a public dataset show that the proposed method in this paper outperforms other baseline methods.

## 1. Introduction

With the vigorous development of artificial intelligence, more and more companies are introducing AI into the operation and maintenance field. The concept of artificial intelligence for IT operations (AIOps) came into being [1]. Compared with the application of artificial intelligence technology in other fields, AIOps has the necessary elements for a certain vertical field of artificial intelligence technology. First, this concerns the actual application scenarios. Almost all key technologies in AIOps are inseparable from machine learning algorithms. Second, it involves massive amounts of data. The enterprise’s operation and maintenance monitoring platform generates massive amounts of monitoring indicator data and operation and maintenance logs constantly. Third, it involves a large number of annotations. Since operation and maintenance personnel are domain experts themselves, a large amount of labeled data will be generated in their daily work. Massive data and annotations lower the threshold for researching machine learning algorithms, which is beneficial to the rapid progress of algorithm research. Therefore, AIOps has broad prospects in the field of machine learning and is worthy of the attention of researchers in the field of machine learning.

KPI (key performance indicator) refers to the monitoring indicators (such as delay, throughput, etc.) for operation and maintenance objects such as services and systems. Its storage form is also known as time series. KPI anomaly detection is an underlying core technology of AIOps. It aims to discover abnormal behaviors in the software and hardware of internet services by analyzing KPI curves, such as increased access delays, network device failures, and sharp reductions in access users, etc. [2]. KPI anomaly detection can provide a decision-making basis for subsequent alarm, automatic stop loss, root cause analysis, etc. In the actual scenes, due to the scarcity of outlier data, various types of anomalies, and various types of KPI, it brings great challenges to anomaly detection.

At present, the industry and academia have proposed a series of anomaly detection algorithms, such as statistical method, prediction method, supervised learning method, unsupervised learning method, and deep generation model method. The most commonly used statistical method is the k-sigma [3] theory, assuming that the data obey a normal distribution, and the data with a small probability of occurrence are regarded as an outlier. However, the actual data distribution does not always follow a normal distribution. The basic idea of the prediction method is to determine whether it is abnormal by comparing the difference between the predicted value and the real value. This method mainly includes the traditional time series prediction models ARIMA [4], LSTM [5], and Holter–Winter [6]. The method based on supervised machine learning can avoid parameter adjustment in traditional statistical algorithms, such as the scalable general anomaly detection framework EGADS [7] proposed by Yahoo and the integrated supervision method Opprentice [8] proposed by Tsinghua NetMan Lab. Although machine learning methods can achieve higher accuracy, the disadvantage is that they require a great deal of manual annotation, covering comprehensive data types and abnormal conditions, which is extremely difficult to achieve in actual scenes [9]. Anomaly detection based on unsupervised learning and deep generative models has become more and more popular in recent years, such as one-class SVM [10], GMM [11], VAE [12,13,14], and CVAE [15]. The deep generative model can more fully capture the complex features of the data and has higher accuracy, so it has received extensive attention.

As a typical representative of the deep generative model, VAE has achieved good results in KPI anomaly detection. However, it does not consider the time dependence of the data, which limits its applicability to time series. Moreover, there is only one fixed-length vector between the encoder and decoder, which leads to its limitations. This fixed-length vector cannot fully store all the related information in the sequence, and it needs to pay attention to the relevant parts of the sequence selectively. In addition, whether VAE uses reconstruction error or reconstruction probability as anomaly scores, a threshold must be set to obtain a more accurate result. However, for massive different types of KPIs, it is difficult to set thresholds uniformly.

Based on the above analysis, this paper proposes a new network structure (DA-LSTM-VAE) that combines attention mechanism, LSTM, and VAE and applies it to KPI anomaly detection. This method aims to automatically discover anomalies in KPI time series, such as abnormal points or abnormal intervals, and then notify operations engineer through alarms. The main contributions of this paper are as follows:

(1) An improved VAE network structure is proposed. The traditional neurons in the encoder and decoder of VAE are replaced by LSTM, which can better extract the time dependence of KPI data. In addition, LSTM-VAE can automatically extract the potential features of KPI data, avoiding the dependence on manual feature extraction;
(2) The dual-stage attention mechanism is introduced in LSTM-VAE for the first time, and the new model structure is named DA-LSTM-VAE. In the first stage, attention is weighted to the original KPI time series. In this way, important input sequences can be highlighted, thereby achieving the effect of denoising. In the second stage, attention is weighted to latent variables of LSTM-VAE. In this way, important latent variable representations across all time steps can be adaptively selected to capture the long-term dependence of KPI time series;
(3) In the anomaly detection stage, an adaptive threshold method is proposed. The reconstruction probability is taken as the anomaly score. Reconstruction probability is a probability measure considering the variability of variable distribution, which is more objective than reconstruction error. The possible fluctuation range of current anomaly scores is judged according to historical anomaly scores so as to achieve the effect of adaptive threshold.

## 2. Related Work

### 2.1. Anomaly Detection Based on VAE

The variational autoencoder (VAE) [16] is an unsupervised generative network model consisting of an encoder and decoder. The encoder is used to learn the distribution of training data and generate compression values of training data, and the decoder reconstructs the compressed data. An et al. [12] proposed an anomaly detection method based on VAE and introduced a novel probabilistic anomaly score that takes into account the variability of the data. Kawachi et al. [13] proposed a new abnormal probability representation method, which defined the normal and anomaly distributions using the analogy between a set and the complementary set. Finally, these distributions are applied to the unsupervised VAE. Xu et al. [14] proposed donut and applied the deep generative model to KPI anomaly detection for the first time. Donut puts forward innovations such as M-ELBO and MCMC iteration based on VAE, which has excellent performance on periodic KPIs. Li et al. [17] proposed ACVAE, a KPI anomaly detection algorithm through active learning and contrast-VAE-based detection models. The out-of-band information (including background information and feedback information) is integrated in VAE, so it has a better detection effect. Zhang et al. [18] proposed an unsupervised KPI anomaly detection approach, named AnoTransfer, by combining a novel VAE-based KPI clustering algorithm with an adaptive transfer learning strategy. VAE can jointly train a basic model by clustering KPI fragments with similar shapes. The performance of the anomaly detection model is improved by using KPI with a similar shape in transfer learning. However, VAE does not consider the time dependence of the data, thus limiting its applicability to time series. Therefore, introducing LSTM into VAE can extract the time dependence and correlation of data to obtain higher data reconstruction accuracy.

### 2.2. Anomaly Detection Based on LSTM-VAE

If the operation and maintenance data detection is regarded as a Seq2Seq [19] problem, the sequence can be understood as time series. Encoder–decoder is a widely used architecture in the Seq2Seq problem. Malhotra et al. [20] proposed an encoder–decoder based on LSTM. The encoder learns the vector representation of input time series, and the decoder uses this representation to reconstruct time series. Then, the reconstruction error is used to detect anomalies. Daehyung et al. [21] proposed a VAE based on LSTM, which can preferably capture the time dependence of data. In addition, a state-based threshold is introduced to increase detection sensitivity and reduce false-alarm rates. Lin et al. [22] combined VAE and LSTM. The VAE encoder first extracts the features of input data and then inputs extracted features into LSTM network for fault detection or classification. Su et al. [23] combined VAE and GRU. GRU captures the complex time dependence between multivariate observations in space, and VAE maps observations to random variables. In the phase of anomaly detection, this method judges entity anomalies by several univariate time series with low reconstruction probability. Niu et al. [24] proposed LSTM-based VAE-GAN for time series anomaly detection. The anomaly score consists of the reconstruction error of VAE and the discrimination results of the discriminator, which makes it more able to distinguish anomalies from normal data. Chen et al. [25] proposed a joint model predictor anomaly detector (PAD). The prediction block (LSTM) takes clean input from the reconstructed time series by VAE, which makes it robust to the anomalies and noise for prediction task. In the meantime, the LSTM block maintains the long-term sequential patterns, which are out of the sight of a VAE encoding window. This leads to the better performance of VAE in anomaly detection than when it is trained alone. Chen et al. [26] proposed a LSTM-based semi-supervised VAE anomaly detection strategy called LR-SemiVAE. By introducing an LSTM network into the VAE encoder and decoder, the model can fully learn the time dependence of KPI time series. However, the biggest limitation of the encoder–decoder model is that the only connection between the encoder and decoder is a fixed-length vector, which cannot fully store all relevant information in the sequence. It is necessary to selectively pay attention to the relevant parts of sequence to improve reconstruction performance, so attention mechanism is introduced into encoder–decoder [27]. In addition, traditional attention mechanism is only used in the input stage of the decoder [28,29,30]. Yao et al. [31] also introduced an attention mechanism in the input stage of the encoder. In this way, the encoder can focus on the important driving factors of input data instead of treating all factors equally. Therefore, this paper introduces attention mechanism in both the encoder and decoder input stage of LSTM-VAE. 

## 3. Anomaly Detection Method

### 3.1. Method Flow

**Figure 1:**
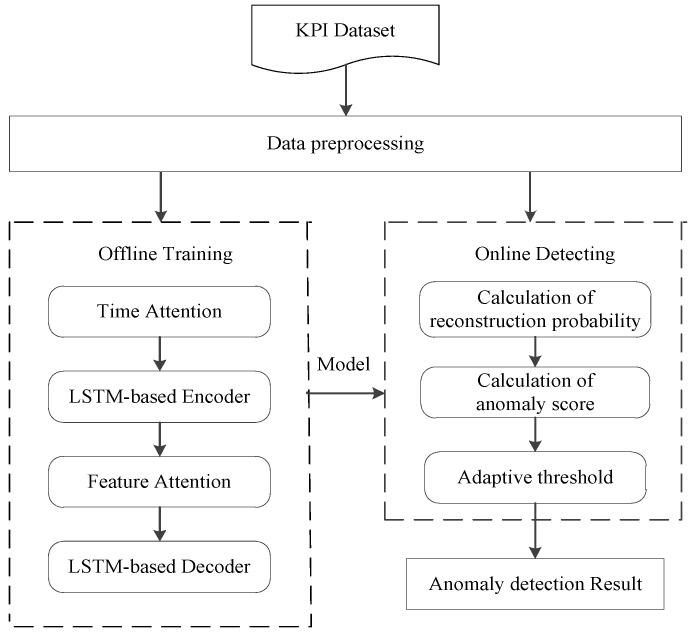
Method flow chart.

The flow of anomaly detection method proposed in this paper is shown in Figure 1, which includes two modules: offline training and online detecting. Data preprocessing is the common module of two modules, which mainly standardizes the data and fills in missing values. After data preprocessing, the data are input to the offline training module for learning, thereby capturing the normal pattern of the data sequence. The offline training module uses historical data batch processing to train the model. The whole process minimizes the loss function so that the model is optimal. According to the trained model, the online detecting module calculates the reconstruction probability of new observation value after preprocessing and obtains anomaly scores. Then, the adaptive threshold is used to determine whether anomaly scores are within the normal fluctuation range. Anomaly detection results are finally output. 

In order to make the method in this paper easier to understand, the main symbols in this paper are summarized as shown in Table 1.

### 3.2. Data Preprocessing

At present, most methods focus on the discussion of algorithms and ignore the necessity of data preprocessing. In the real world, data are usually incomplete, inconsistent, and highly susceptible to noise. In order to eliminate the influence of data on the algorithm, the missing value and standardization of data are firstly processed in this paper.

#### 3.2.1. Missing Value Processing

Data loss occurs in the process of data transmission, sampling, and recording. Therefore, it is necessary to preprocess time series before establishing time series model. The processing of missing values in a time series is an important part of preprocessing. Improper processing will result in a large number of errors and seriously affect results. Traditional missing-value processing methods include zero value or mean value replacement. The missing-value processing method in ref. [14] is adopted in this paper. This method fills missing points with 0 instead of using some existing algorithms. Since VAE is a generating model, it can generate data and fill in missing values. Here, we assume that the complete data are represented as $X=\{X_{obs},X_{mis}\}$, where $X_{obs}$ represents observable data, and $X_{mis}$ represents missing data. The input sample X is transformed from $\left\{X_{obs},X_{mis}\right\}$ to $\left\{X_{obs},{X_{mis}}^{\prime }\right\}$ after reconstruction, that is, the reconstructed sample $X^{\prime }$. $X^{\prime }$ is the input of MCMC [32] iterative. According to Bayes’ theorem, the posterior probability of missing data under the condition of observed data can be obtained. As shown in Formula (1),

(1) $$P({X_{mis}}^{\prime }|X_{obs})=\int f(X_{obs}|{X_{mis}}^{\prime },\alpha )k(\alpha |X_{obs})d\alpha ,$$

where $k(\alpha |X_{obs})$ is the posterior probability of α under the observable data, but the above formula cannot be directly integrated. $P({X_{mis}}^{\prime }|X_{obs})$ is simulated by MCMC method, and then, ${X_{mis}}^{\prime }$ of the time series was randomly extracted from it.

#### 3.2.2. Data Standardization

Data standardization (normalization) processing is a basic work of data mining. Different evaluation indicators often have different dimensions and dimension units, which will affect the results of data analysis. In order to eliminate the dimension impact between indicators, data standardization processing is required to solve the comparability between data indicators. After the original data re standardized, all indicators are in the same order of magnitude, which is suitable for comprehensive comparative evaluation. This paper uses the maximum–minimum normalization method to process the KPI data so that the data are distributed between 0–1. As shown in Formula (2),

(2) $$x^{*}=\frac{x-\min}{\max-\min},$$

### 3.3. Offline Training

Offline training aims to provide a model for online detecting, and its model is LSTM-VAE based on dual-stage attention. The attention mechanism is introduced in both the encoder and decoder input stages of LSTM-VAE to highlight important time points and features, and considering the disappearance of *KL* divergence during model training, the model is optimized by minimizing the loss function.

#### 3.3.1. Dual-Stage Attention-Based LSTM-VAE (DA-LSTM-VAE) Model

Offline training aims to provide a model for online detecting, and its model is LSTM-VAE based on dual-stage attention. The attention mechanism is introduced in both the encoder and decoder input stages of LSTM-VAE to highlight important time points and features, and considering the disappearance of *KL* divergence during model training, the model is optimized by minimizing the loss function. The dual-stage model includes both the early stage of stimulus selection and the later stage of stimulus selection. The first stage selects the primary stimulus characteristics, and the second stage uses the classification information to decode the stimulus [33]. The dual-stage attention mechanism can select the relevant driving sequence and enhance the long-term dependence of time series [31,34]. This paper names the dual-stage attention as time attention and feature attention, respectively. In this paper, a sliding window with a certain step length is used to divide the time series into sub-sequences, which correspond to the input variables. The time attention is used to weight the original data sample x to obtain the updated time series $\tilde{x}$. $\tilde{x}$ is encoded as latent variables z. z is transformed into context variable c by the feature attention. c is decoded to output the reconstructed data sample y.

**Figure 2:**
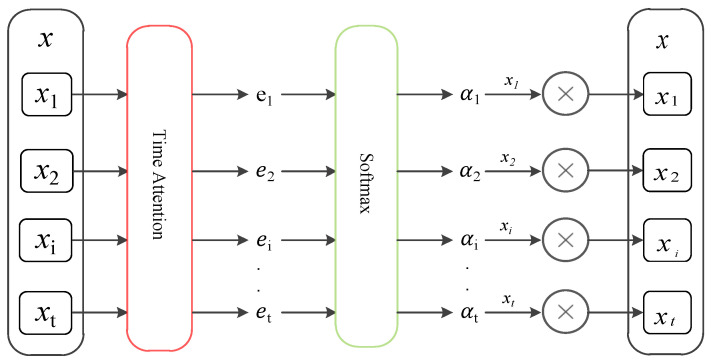
Attention-mechanism-weighted original time series.

Time attention weights original time series to highlight important input sequences. The process is shown in Figure 2.

The encoder used in this paper is based on LSTM neural network units [35]. In the time attention phase, it is necessary to calculate the hidden layer state $h_{t}$ of encoder at the current moment based on input $x_{t}$ at the current moment and the hidden layer state $h_{t-1}$ of encoder at the previous moment. The calculation process is shown in Formula (3).

(3) $$h_{t}=\mathrm{LSTM}(h_{t-1},x_{t}),$$

LSTM is a special RNN, which can solve the long-term dependency problem well. The state-update process of the LSTM unit is shown as follows:

(4) $$f_{t}=\sigma (W_{f}\cdot [h_{t-1},x_{t}]+b_{f}),$$

(5) $$i_{t}=\sigma (W_{i}\cdot [h_{t-1},x_{t}]+b_{i}),$$

(6) $${\tilde{C}}_{t}=\tanh(W_{C}\cdot [{h_{t}}_{-1},x_{t}]+b_{C}),$$

(7) $$C_{t}=f_{t}*C_{t-1}+i_{t}*{\tilde{C}}_{t},$$

(8) $$O_{t}=\sigma (W_{o}[h_{t-1},x_{t}]+b_{o}),$$

(9) $$h_{t}=O_{t}*\tanh(C_{t}),$$

where $f_{t},i_{t},{\tilde{C}}_{t},C_{t},O_{t}$ represent the forgetting gate, input gate, candidate memory unit state, memory unit, and output gate, respectively. $W_{f},W_{i},W_{o},W_{c}$ represent the weight matrix of corresponding gate and memory unit, respectively; $b_{f},b_{i},b_{o},b_{c}$ represent the corresponding gate and memory unit bias items, respectively. σ represents the sigmoid activation function.

Next, the attention mechanism is constructed through hidden layer state $h_{t-1}$ and unit state $C_{t-1}$ of the encoder at the previous moment.

(10) $$e_{t}=v_{e}^{T}\tanh(W_{e}[h_{t-1};C_{t-1}]+U_{e}x+b_{e}),$$

(11) $$a_{t}=\frac{\exp(e_{t})}{\sum_{i=1}^{t}\exp(e_{i})}$$

Among them, [∗;∗] is an aggregation operation. $v_{e}$, $W_{e}$, $U_{e}$, and $b_{e}$ are learnable network parameters. The attention score $e_{t}$ depends on the current input and historical hidden state. After $e_{t}$ is normalized by the SoftMax() function, the attention weight coefficient $a_{t}$ is obtained. In the time attention stage, this paper only aims to obtain the weight of attention at different moments, so it does not carry out the weighted summation operation. The updated time series can be expressed as

(12) $$\tilde{x}=(\alpha_{1}x_{1},\alpha_{2}x_{2},\ldots ,\alpha_{t}x_{t}),$$

**Figure 3:**
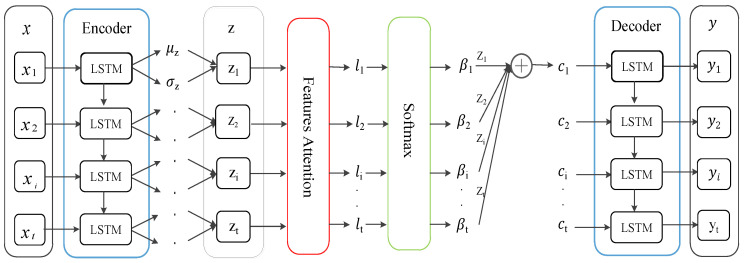
The attention mechanism optimizes latent variables of LSTM-VAE.

Feature attention assigns different weights to the latent space representation of time series to highlight important features. The process is shown in Figure 3. At this stage, the attention mechanism is used to optimize the latent variables of LSTM-VAE. First, updated time series $\tilde{x}$ are input to the LSTM-based encoder, which estimates the mean $\mu_{z}$ and variance $\sigma_{z}$ of latent variables z by two linear modules to approximate the posterior $q_{\varphi }(z_{t}|\tilde{x})$. Then, the reparameterization operation is carried out to obtain latent variables z; that is, $z=\mu_{z}+\sigma_{z}⊙\varepsilon$, where ε∼N(0,1), and ⊙ represents the product at the element level. The latent variable z is transformed into the context vector c by the feature attention. Finally, the LSTM-based decoder outputs reconstructed time series y through the generation network $p_{\theta }(y_{t}|c_{t})$.

Different from the first stage, the attention mechanism at this time is based on the previous hidden layer state $d_{t-1}$ and unit state $C_{t-1}^{\prime }$ of the decoder. The calculation process is as follows:

(13) $$l_{t}=v_{l}^{T}\tanh(W_{l}[d_{t-1};C_{t-1}^{\prime }]+U_{l}z+b_{l}),$$

(14) $$\beta_{t}=\frac{\exp(l_{t})}{\sum_{j=1}^{t}\exp(l_{j})}$$

Among them, [∗;∗] is an aggregation operation. $v_{l}$, $W_{l}$, $U_{l}$, and $b_{l}$ are learnable network parameters. The attention score $l_{t}$ depends on current input $z_{i}$ and historical hidden states $d_{t-1}$ and $C_{t-1}^{\prime }$ of the decoder. After $l_{i}$ is normalized by the SoftMax() function, the attention weight coefficient $\beta_{i}$ is obtained. It expresses the importance of latent variable $z_{i}$ to the output result so that important features are taken into consideration when outputting. The context vector $c_{t}$ is the weighted sum of all latent variables $\left\{z_{1},\;z_{2},\;\ldots ,\;z_{t}\right\}$. The calculation formula is as follows:

(15) $$c_{t}=\sum_{i=1}^{t}\beta_{i}z_{i},$$

After the context vector $c_{t}$ is obtained, the conditional generation distribution can be calculated according to $p_{\theta }({\tilde{x}}_{t}|c_{t})$. If its log likelihood $\ln[p_{\theta }({\tilde{x}}_{t}|c_{t})]$ in the conditional generation distribution is larger, the effect of reconstruction is better.

#### 3.3.2. Loss Function

In order to solve the problem of overfitting in AE [36] training, VAE adds *KL* loss to the loss function. It measures the distance between approximate posterior distribution $q_{\theta }(z_{t}|{\tilde{x}}_{t})$ and true posterior distribution $p_{\theta }(z_{t})$. The objective of VAE’s loss function optimization is to maximize the likelihood function of the generated data and minimize the *KL* divergence of the approximate posterior distribution to the true posterior distribution. Since this paper uses the attention mechanism to measure the importance of latent variable $z_{t}$, the context vector $c_{t}$ is obtained. Therefore, the improved loss function is shown in Formula (16).

(16) $$L_{avae}=\lambda_{KL}[D_{KL}(q_{\varphi }(c_{t}|{\tilde{x}}_{t})||p_{\theta }(c_{t}))]-E_{q_{\varphi }(c_{t}{|\tilde{x}}_{t})}[\log p_{\theta }({\tilde{x}}_{t}|c_{t})],$$

It can be seen that the loss function consists of two parts: reconstruction loss and *KL* loss. However, when VAE and LSTM are trained together, the problem of *KL* divergence disappears. This paper uses the *KL* cost-annealing method proposed in [37]. This method multiplies the *KL* term by a weight coefficient $\lambda_{KL}$. The coefficient size is 0 at the beginning of training and gradually increases to 1 as the number of training increases. Thus, the reconstruction term is paid attention to in the early stage of training.

### 3.4. Online Detecting

A trained model is used to determine whether the observed value of a time step is abnormal during online detecting. This paper uses normal data to train the model and extracts the normal pattern of data. When the data contain anomalies, they cannot be reconstructed well; that is, it has a low reconstruction probability. Then, reconstruction probability is used as anomaly scores. The higher scores, the more likely that it is an abnormality. Finally, adaptive threshold is applied to anomaly scores to obtain the anomaly detection results.

#### 3.4.1. Calculation of Reconstruction Probability

This paper adopts the method proposed in [12], using reconstruction probability as anomaly scores. The reconstruction probability is calculated by random hidden variables that can output the parameters of the distribution of original input variables rather than the input data. Therefore, it is more principled and objective than reconstructing errors. It uses Monte Carlo to estimate the reconstruction item of VAE. The calculation formula is as follows:

(17) $$E_{q_{\varphi }(c_{t}{|\tilde{x}}_{t})}[\log p_{\theta }({\tilde{x}}_{t}|c_{t})]\approx \frac{1}{L}\sum_{l=1}^{L}\log p_{\theta }({\tilde{x}}_{t}|\mu_{c}(t,l),\sigma_{c}(t,l)),$$

The calculation process can be described as: First, the original data are passed through the encoder to obtain the distribution $\mu_{z}$ and $\sigma_{z}$ of data, and latent variable z is obtained by sampling in the distribution. The latent variables z are transformed into context vectors c by the attention layer. The decoder outputs parameters $\mu_{c}$ and $\sigma_{c}$ of context vector c. These parameters are used to calculate the probability of generating original data from the distribution. For each data c(t,l) in the extracted sample L, loop about l. After the loop is over, multiple $\mu_{c}(t,l),\sigma_{c}(t,l)$ can be obtained, that is, multiple c. Finally, the log likelihood of the input data ${\tilde{x}}_{t}$ is calculated on the approximate posterior distribution of c, and the average reconstruction probability on all samples c is calculated.

#### 3.4.2. Calculation of Anomaly Score

Anomaly scores are negative reconstruction probabilities. If the sample has a lower reconstruction probability, it will have a higher anomaly score. The reconstruction probability $E_{q_{\varphi }(c_{t}{|\tilde{x}}_{t})}[\log p_{\theta }({\tilde{x}}_{t}|c_{t})]$ can be calculated immediately at time t. By taking the additive inverse of reconstruction probability, anomaly scores can be obtained. The calculation formula is as follows:

(18) $$S_{t}=-E_{q_{\varphi }(c_{t}{|\tilde{x}}_{t})}[\log p_{\theta }({\tilde{x}}_{t}|c_{t})],$$

#### 3.4.3. Adaptive Threshold

After calculating anomaly scores, it is often necessary to set a threshold to determine whether it is an anomaly. Adjusting different values will change the detection rate and false-alarm rate accordingly. The raw anomaly scores represent an instantaneous measure of the predictability of current input data, which is suitable for predictable scenes. However, in many practical applications, the underlying system is inherently noisy and unpredictable. For example, the latency indicator of a website is usually low, but occasionally, random jumps and corresponding peaks in anomaly scores are not uncommon. Anomalies usually occur continuously in actual application scenes. However, setting a fixed threshold for original anomaly scores can lead to many false positives and false negatives. Therefore, this paper proposes an adaptive threshold method to reduce false alarms as much as possible. The adaptive threshold can be defined as:

(19) $$[S_{t}-\sigma ,S_{t}+\sigma ],$$

Among them, σ is the floating range of anomaly scores $S_{t}$. Considering the time relevance of anomaly scores, we calculate the possible fluctuation range of anomaly scores at the current moment based on historical anomaly scores [38]. This paper uses standard deviation σ to describe the range of anomaly scores, which is defined as follows:

(20) $$avg=\sum_{i=t-w+1}^{t}\frac{S_{i}}{w},$$

(21) $$V=\sqrt{\sum_{i=t-w+1}^{t}\frac{{\left(S_{i}-avg\right)}^{2}}{w}},$$

(22) σ=ρ·V,

Among them, w is the size of the sliding window. avg is the average of historical anomaly scores. V is the standard deviation. ρ is the standard deviation coefficient. Therefore, anomaly is determined based on whether anomaly score at the current moment is within normal fluctuation range:

(1) Normal: $S_{t}-\sigma \leq S_{t}\leq S_{t}+\sigma$;
(2) Abnormal: $S_{t}<S_{t}-\sigma$ or $S_{t}>S_{t}+\sigma$.

## 4. Experiment

This section verifies the validity of this method by comparing it with different methods in a public dataset. In the environment of Python 3.6, we used Keras to implement the method in this paper and used Tensorflow as the backend engine. The hyperparameters in the experiment are set as follows: the time step size is 60, the hidden layer size of LSTM is 128, the latent variable dimension is 10, the batch size is 256, the number of iterations is 300, Adam is selected as the optimizer, and the initial value of learning rate is 0.0005.

### 4.1. Dataset

**Figure 4:**
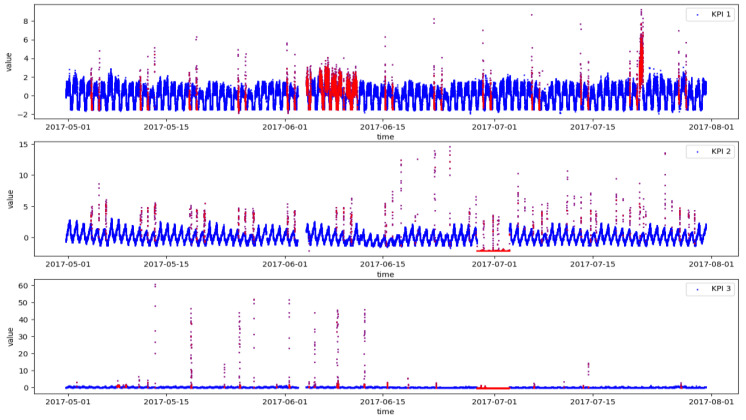
Three KPIs from real production environments (Blue represents normal and red represents abnormal).

This paper uses the dataset of AIOps Challenge held by Tsinghua University in 2018 (http://iops.ai/competition_detail/?competition_id=5&flag=1 (accessed on 10 September 2021)). According to the organizer’s description, the data are collected from the real operation and maintenance environment of top internet companies such as Sogou, Tencent, eBay, Baidu, and Alibaba. There are 28 KPIs in the dataset, including service KPIs that can reflect the scale and quality of web services, such as web response time, web page visits, and connection errors. It also includes machine KPIs that can reflect the health status of machines (servers, routers, switches), such as CPU usage, memory usage, disk IO, network card throughput, etc. We randomly selected three KPIs from them to conduct experiments. The details of the dataset are shown in Table 2. The data sampling interval is one minute, and sampling time is 91 days. It can be found that the positive and negative ratios of data are extremely unbalanced, and anomaly ratios are both less than 10%. Figure 4 shows the visualization of three KPIs. It can be seen that the curves of KPI 1 and KPI 2 exhibit a certain periodicity, while the curve of KPI 3 is stable and non-periodic.

### 4.2. Baseline and Evaluation Metrics

In order to evaluate the performance of the method in this paper, we implemented three baseline methods. Three baselines that also use VAE as the basic structure were selected for comparative experiments. The hyperparameters of all methods are the same in the experiment:

- VAE [14]: Donut is an unsupervised anomaly detection method based on VAE. Through the improved variational lower bound and Markov chain Monte Carlo interpolation technology, donut can be used without labels. During anomaly judgment, it scores anomalies on the data through the reconstruction probability and then determines anomalies according to the set threshold value;
- LSTM-VAE [21]: This method proposes a long–short-term memory-based variational autoencoder (LSTM-VAE), which can extract the time dependence of sequence data and achieve better performance than donut in KPI anomaly detection with partial time dependence. In addition, for the problem that sometimes non-anomalous has a higher anomaly score, this method introduces a state-based threshold, which can reduce false alarms and increase sensitivity by changing anomaly scores;
- LSTM-VAE-Attention [30]: This method introduces an attention mechanism based on a variational recurrent autoencoder. It uses a variational self-attention mechanism (VSAM) to improve the performance of encoding–decoding processes. Finally, the reconstruction probability is used as anomaly scores to detect anomaly patterns.

This paper uses Precision, Recall, F1-score as evaluation metrics. Precision represents the proportion of correct prediction being positive to total prediction being positive. Recall represents the proportion of correct prediction being positive to total actual being positive. F1-score is the weighted harmonic average of Precision and Recall. The calculation formula is as follows:

(23) $$Precision=\frac{TP}{TP+FP},$$

(24) $$Recall=\frac{TP}{TP+FN},$$

(25) $$F1\text{-}score=\frac{2\times Precision\times Recall}{Precision+Recall},$$

where TP is the number of anomaly points correctly detected, FP is the number of normal points incorrectly identified as anomaly points, and FN is the number of anomaly points incorrectly identified as normal points.

### 4.3. Experimental Results and Comparative Analysis

**Figure 5:**
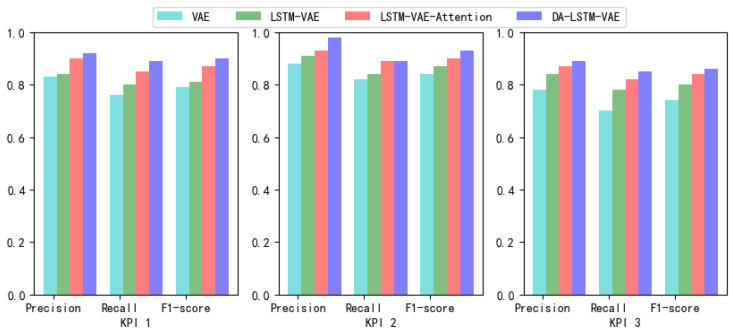
Data visualization effect in Table 3.

This paper uses a 10-fold method to test the effectiveness of four methods. The best Precision, Recall, and F1-score of four methods are shown in Table 3. It can be seen that four methods perform well in periodic KPI 1 and KPI 2. However, VAE performs poorly in non-cyclical KPI 3. LSTM-VAE takes into account the time correlation of sequence, so it has higher accuracy than VAE. LSTM-VAE-Attention uses attention mechanism to pay attention to important latent variables, so the effect is slightly improved than LSTM-VAE. DA-LSTM-VAE in this paper obtained an F1-score in the range of 0.85–0.95, which is better than other methods. We use the data histogram to visualize data in the table, as shown in Figure 5.

**Figure 6:**
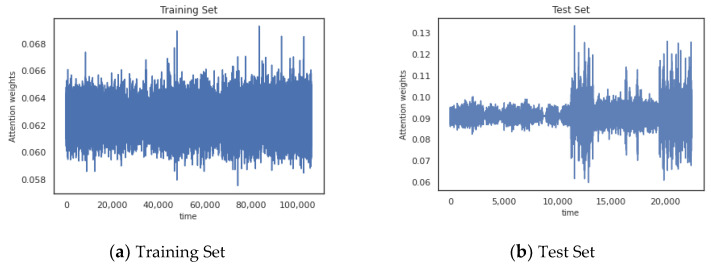
The attention mechanism assigns different weights to different moments in time series.

Before time series are entered into the encoder, different weights are assigned to different time points in time step using the attention mechanism. Similar patterns can be observed for other time steps. Figure 6a shows the weight of attention at each time point in the training set. Figure 6b shows the weight of attention at each time point in the test set.

It can be observed that since the training set does not contain anomalies, the attention weights fluctuate within a stable interval. Time points in the test set that contain anomalies are assigned a larger weight, which leads to significant fluctuations.

**Figure 7:**
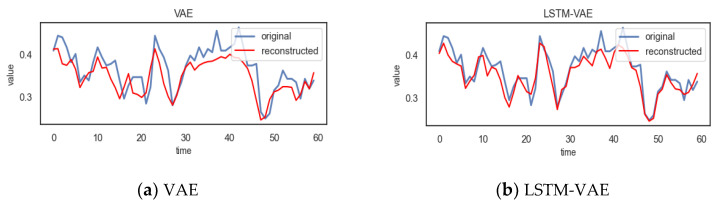

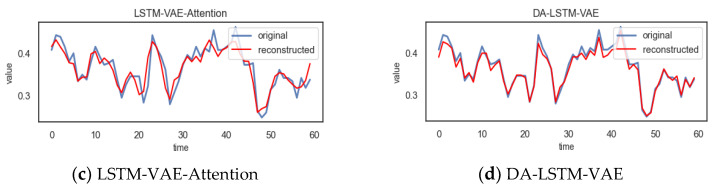
The reconstruction effect of each method in a time step on a normal time series.

DA-LSTM-VAE uses normal data for training, thereby learning the normal pattern of the data. In this way, the model can reconstruct normal data sequences well. In order to prove that our model can effectively improve the encoding–decoding process and enhance the reconstruction effect, as shown in Figure 7, the reconstruction effect of each method on a time step of a normal time series was compared.

It can be observed from Figure 7 that the reconstruction value of DA-LSTM-VAE on a normal time series is closer to the true value; that is, the reconstruction effect is better than other methods.

**Figure 8:**
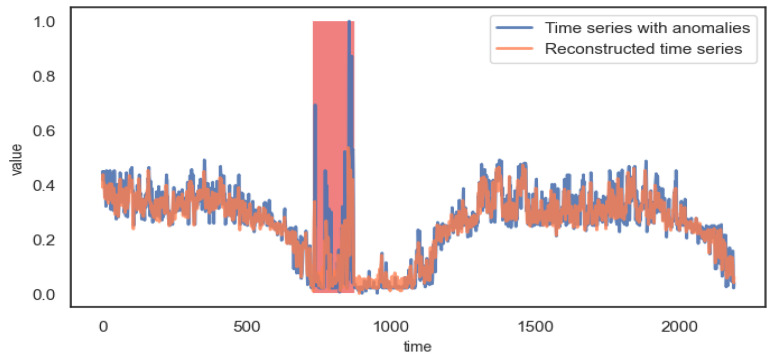
Reconstruction effect of anomaly time series.

When the time series contains anomalies, the anomaly series will not be reconstructed well. In order to observe this phenomenon intuitively, Figure 8 shows the comparison between time series with anomalies and reconstructed time series.

It can be observed from Figure 8 that the anomaly interval of the time series cannot be reconstructed well, so it has a higher reconstruction error and a lower reconstruction probability. Thus, it is possible to detect anomalies.

**Figure 9:**
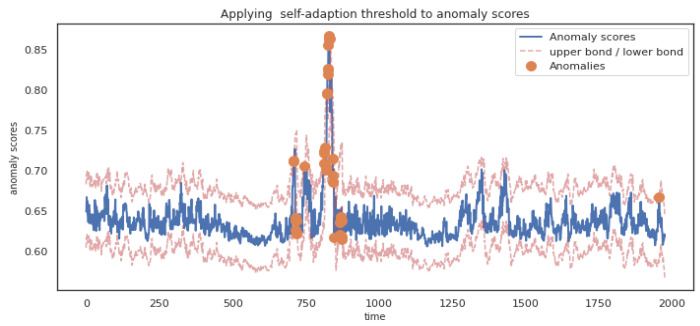
Applying adaptive threshold to anomaly scores.

During anomaly judgment, this paper uses reconstruction probability as anomaly scores. Setting a fixed threshold directly on anomaly scores will not only lead to a large number of false positives and false negatives but also need to adjust the threshold manually. Therefore, this paper proposes an adaptive threshold method for the above problems, which judges the possible fluctuation range of anomaly scores at the current moment based on historical anomaly scores. The effect of anomaly judgment on anomaly scores through the adaptive threshold is shown in Figure 9.

It can be seen from Figure 9 that if anomaly scores exceed the upper or lower bound of threshold, they will be judged as anomaly.

**Figure 10:**
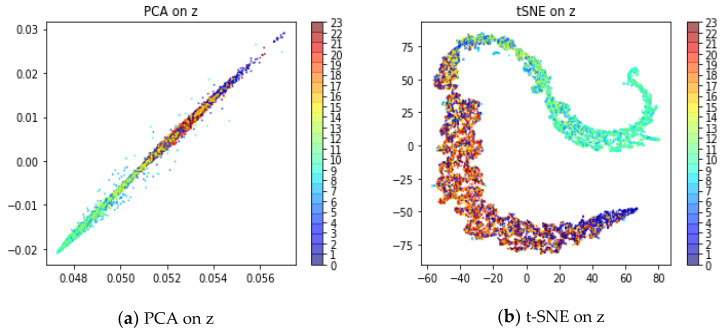
Time correlation of latent variable *z*.

In this paper, LSTM is used as the encoder and decoder of VAE, which can better capture the time correlation of sequence data. In the experiment, the test set containing some anomalies was selected to verify the effect of time correlation. The hour of the time series was extracted as the label; that is, there are 24 types of labels. Principal component analysis (PCA) [39] and *t*-distributed stochastic neighbor embedding (*t*-SNE) [40] are used to reduce the dimension of latent variables to two dimensions for visualization. Figure 10a shows the visualization of PCA. Figure 10b shows the visualization of *t*-SNE.

The visualization effect shows that the time series present the phenomenon of color gradients in latent variables space. The model maps the sequence aligned in time to the same region of latent variable space. In other words, the model captures the temporal correlation of sequence. However, anomalous moments present a large deviation, such as 8 o’clock.

**Figure 11:**
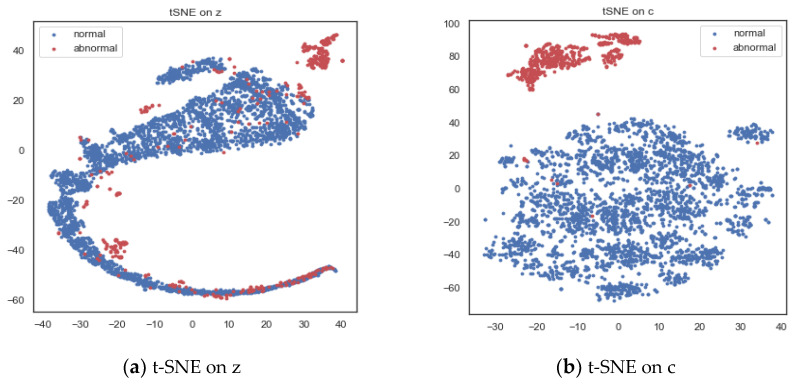
Two-dimensional visualization of latent variable *z* and context vector *c*.

To highlight the advantages of introducing attention mechanism, we also selected part of the test set. T-SNE is used to visualize latent variable *z* and context vector *c*, respectively. Figure 11a shows the two-dimensional representation of latent variable *z*. It can be found that the difference between normal data and abnormal data in latent variable space is not very obvious, and there is more overlap. Figure 11b shows the two-dimensional representation of context vector *c*. It can be found that normal data and abnormal data are well distinguished. This demonstrates the use of attention mechanism to optimize latent variables, and a better potential representation is obtained.

## 5. Conclusions

In this paper, we proposed a novel dual-stage attention-based LSTM-VAE (DA-LSTM-VAE) for KPI anomaly detection. In this method, LSTM is used as the encoder and decoder of VAE to capture the time dependence and correlation characteristics of time series. Dual-stage attention consists of time attention in the input stage of encoder and feature attention in the input stage of the decoder. Time attention weights original time series to highlight important time points. Feature attention optimizes the latent variable representations of LSTM-VAE to highlight important features. Through the above improvements, our method can improve the encoding and decoding process, better capture the long-term dependence of time series, and learn better potential representations. In addition, this paper proposes an adaptive threshold method, which can greatly reduce false positives and false negatives and guarantee the accuracy of KPI anomaly detection. Experiments based on KPI time series data show that the KPI anomaly detection method proposed in this paper has a better detection effect than baseline methods. If the method in this paper is applied to the real environment, it includes two steps of offline training and online detection. The offline training step is to learn the normal mode of data, and the online detection step is to detect anomalies according to the trained model. By monitoring the KPI time series in real time, abnormal time points can be automatically discovered so that operations engineer can handle them in time and ensure the smooth operation of the application.

## Figures and Tables

**Table 1:** Main symbols table.

Symbolic Representation	Symbolic Interpretation
$x_{t}$	KPI time series after data preprocessing
$h_{t}$	LSTM hidden layer state
$e_{t}$	Attention scores at the time attention stage
$a_{t}$	Attention weights at the time attention stage
${\tilde{x}}_{t}$	KPI time series obtained through time attention stage
$\mu_{z}$	Mean value estimated by encoder
$\sigma_{z}$	Variance estimated by encoder
ε	Auxiliary noise variables
$z_{t}$	Latent variables obtained by reparameterization
$q_{\theta }(z_{t}|{\tilde{x}}_{t})$	Approximate posterior distribution
$p_{\theta }(z_{t})$	True posterior distribution
$l_{t}$	Attention scores at the feature attention stage
$\beta_{t}$	Attention weights at the feature attention stage
$c_{t}$	Context variables obtained through feature attention stage
$y_{t}$	Reconstructed KPI time series
$L_{avae}$	Loss function of the model
$\lambda_{KL}$	Weight coefficient of *KL* loss
$S_{t}$	Anomaly scores for KPI time series

**Table 2:** Description of three KPIs in dataset.

Data Set	KPI 1	KPI 2	KPI 3
Total points	128,562	129,035	129,128
Anomaly points	10,550/8.21%	7666/5.94%	7863/6.09%
Missing points	3233/0.02%	2755/0.02%	2667/0.02%
Duration	91 days	91 days	91 days
Sample frequency	1412.77	1417.97	1418.99

**Table 3:** Anomaly detection results of each method in three KPIs.

Dataset	Method	Precision	Recall	F1-Score
KPI 1	VAE	0.83	0.76	0.79
	LSTM-VAE	0.84	0.80	0.81
	LSTM-VAE-Attention	0.90	0.85	0.87
	DA-LSTM-VAE	0.92	0.89	0.90
KPI 2	VAE	0.88	0.82	0.84
	LSTM-VAE	0.91	0.84	0.87
	LSTM-VAE-Attention	0.93	0.89	0.90
	DA-LSTM-VAE	0.98	0.89	0.93
KPI 3	VAE	0.78	0.70	0.74
	LSTM-VAE	0.84	0.78	0.80
	LSTM-VAE-Attention	0.87	0.82	0.84
	DA-LSTM-VAE	0.89	0.85	0.86

## Data Availability

The data used to support the findings of this study are available from the corresponding author upon request.
